# Adjuvant aromatase inhibitor treatment worsens depressive symptoms and sleep quality in postmenopausal women with localized breast cancer: A one-year follow-up study

**DOI:** 10.1016/j.breast.2022.11.007

**Published:** 2022-11-24

**Authors:** Javier García-Sánchez, Mayra Alejandra Mafla-España, María Dolores Torregrosa, Omar Cauli

**Affiliations:** a Medical Oncology Department, Doctor Peset University Hospital, Valencia, Spain; b Medical Oncology Department, Hospital Center of Wallonie Picarde, Tournai, Belgium; c Frailty Research Organized Group, University of Valencia, Valencia, Spain; d Department of Nursing, University of Valencia, Valencia, Spain

**Keywords:** Aromatase inhibitors, Depression, Cognitive impairment, Breast cancer, Insomnia, AROi, Aromatase inhibitors, GDS, geriatric depression scale, MMSE, Mini-Mental State Examination test

## Abstract

First-line treatment in postmenopausal women with estrogen- and/or progesterone-positive breast cancer consists of aromatase inhibitors (AROi). The ability of AROi to promote or worsen cognitive function, depressive symptoms, sleep quality and performance in basic activities of daily life as primary and concomitant outcomes in long longitudinal studies in post-menopausal women has been seldom investigated. This study is a cohort trial which aimed to determine if there were differences in cognitive function assessment, depressive symptoms, and sleep quality after 1 year under AROi treatment and to determine the interrelations between these symptoms. Methods: A prospective 1-year longitudinal study was performed in a representative sample of tertiary hospital. Women with localized breast cancer newly treated with AROi therapy were evaluated for cognitive functions, depressive symptoms, sleep problems and ability to perform basic activities of the daily life at baseline and after 6 months and 12 months under adjuvant AROi treatment. Results: Analysis of cognitive functions by the Mini-Mental State Examination (MMSE) scores did not show significantly worsening under AROi treatment after 6 months and 12 months of treatment compared to the baseline. Analysis of depressive symptoms with the Geriatric Depression Scale and sleep quality with the Athens Insomnia Scale (AIS) scores showed significant (p < 0.05) changes after 6 and 12 months of treatment with AROi, with women describing more depressive symptoms and more sleep disturbances. Conclusions: Our study found impairments in sleep quality and an increase in depressive symptoms, which has important implications for clinicians as they impair quality of life and adherence to treatment.

## Highlights

• AROi treatment did not worsen cognitive function.
• AROi treatment worsens depressive and insomnia symptoms in those women who had them previously.
• Previous chemotherapy administration and younger age play a role in these effects.

## 1 Introduction

Breast cancer is the most commonly diagnosed cancer in women in the world [1]. Around 80% of invasive breast tumors in postmenopausal women are estrogen-receptor positive breast cancer [2]. Aromatase inhibitors are the first-line adjuvant treatment in postmenopausal patients with estrogen receptor-positive localized breast cancer, being prescribed for at least 5 years and treatment sometimes lasting 10 years [3]. Adjuvant endocrine therapy reduces the annual probability of tumor recurrence by more than one third, regardless of the use of chemotherapy or the age of the patient [4].

Cognitive impairment is the decline of intellectual functions such as thinking, remembering, reasoning and planning, with mild forms of forgetfulness that can lead to severe and debilitating dementia. This is a common process among the elderly. Indeed, age is described as the most important independent risk factor for cognitive impairment and dementia [5]. Estrogens act actively not only in peripheral organs, but also in several areas of the brain involved in cognitive and emotional behavior, and a relationship between reduced estrogen levels and cognitive decline has been proposed in postmenopausal women [6]. However, the relationship between estrogen and cognitive status is complex, and is not only explained by the presence or absence of hormones and their alteration. It has been theorized that the reduced blood concentration caused produced by aromatase inhibitors could induce cognitive deficits [7]. With the increase in survival rates for some types of cancer and a greater interest in the quality of life of cancer survivors, several studies have been published on the complaints and cognitive alterations related to cancer treatment reported by patients [6].

Patients suffering from cancer sometimes present cognitive impairment, also called cancer-related cognitive impairment (CRCI) [8]. Since different studies have used different means of assessment and measurement, its exact incidence and prevalence is unknown, although its prevalence may be as high as 75% [9,10]. Despite the existence of several cohort studies performed with neuropsychological and brain imaging tests, it remains unclear whether cognitive impairment is a consequence of treatment, the cancer itself or psychological factors. In addition, there are other predisposing factors related to CRCI, such as age, genetics, or psychological and sociodemographic factors [11]. Among the oncological treatments implicated in cognitive impairment, surgical treatment, radiotherapy, hormonal treatment, targeted therapy, and immunotherapy have been related to it [8]. Although most studies have focused on breast cancer, CRCI has also been identified in cancer patients with colorectal cancer [12], lung cancer [13], testicular cancer [14], prostate cancer [15], gynecologic malignancies [16]. To facilitate collaborative efforts, in 2011, the International Cognition and Cancer Task Force published consensus recommendations for basic neuropsychological testing for studies of cancer populations that have recently been updated [8,17]. Despite the limited ability to detect changes in cognitive function in post-menopausal women in the studies published to date, there is little evidence that aromatase inhibitors can have a lasting detrimental effect on cognitive performance in breast cancer patients [18]. In addition, factors such as depressive symptoms and their association with the development of cognitive impairment have seldom been taken into account [[19], [20], [21]].

The longitudinal studies on the effects of AROi on cognitive functions in postmenopausal women are limited [6]. In addition to cognitive complaints, women with breast cancer are at higher risk of suffering from a depressive syndrome [[22], [23], [24]], and an increased risk of depression among breast cancer survivors has been reported [[25], [26], [27], [28], [29], [30]]. Some trials have reported an increased risk of depressive symptoms also in patients under AROi [[31], [32], [33]]. However other reports found no significant effects, and a few reports found the opposite, i.e. AROi reduced depressive symptoms at short term [34] and after long-term treatment [35]. The interplay between cognitive function and depressive symptoms is crucial [36,37], and one recent follow-up study have analyzed their interactions [38]. Sleep disturbance is a common complaint among women treated for early-stage breast cancer, with about 70% of breast cancer patients receiving chemotherapy reporting sleep disturbances [39], and up to 85% of patients receiving radiotherapy for breast cancer reporting abnormally frequent nighttime awakenings [40]. The important role of estrogens in sleep regulation is well defined, and a deficiency thereof can cause sleep disturbances such as insomnia [41,42].

This longitudinal study aimed to ascertain whether cognitive function, depressive symptoms, sleep quality and performance in daily activities were affected in patients with localized breast cancer under treatment with aromatase inhibitors after 6 and 12 months of treatment.

## 2 Materials and methods

### 2.1 Study design

In this longitudinal observational study, which evaluated a cohort of women diagnosed with hormone-dependent early breast cancer who had undergone tumor removal surgery, radiotherapy and/or chemotherapy and who were about to begin antiestrogen treatment with AROi to prevent tumoral recurrence. Evaluations were performed before starting hormone treatment, and after six months and one year of treatment with aromatase inhibitors. The trial was conducted in accordance with the guidelines of the Declaration of Helsinki, and the study protocol was approved by the Clinical Research Ethics Committee of the Doctor Peset University Hospital (protocol code57/18, approved on July 5, 2018). The STROBE Statement—Checklist adapted for longitudinal studies was followed.

### 2.2 Sample size

The sample size was determined before the development of the study, For the calculation of the minimum sample size (unicentric study) that was representative of the patients attended to in the Oncology Department of the Hospital Universitario Dr. Peset (Valencia, Spain), it was estimated that an average of 77 post-menopausal women with breast cancer started adjuvant treatment with aromatase inhibitors in the Hospital every year (with the program G*Power 3.1.9.2 (G*Power©, Dusseldorf, Germany). A total of 47 women agreed to participate, and signed the informed consent form before starting the study.

### 2.3 Outcome variables

The Mini-Mental State Examination (MMSE) score was considered as a numerical and categorical variable, which was normal or impaired taking the level of education into account. In addition, the scores of its dimensions (orientation, spatial orientation, immediate recall, attention and calculation, delayed recall, language) were considered independently as numerical variables [43,44]. Given most patients were over 65 years of age, the Yesavage scale (called Geriatric Depression Scale - GDS), which is specific for individuals in this age group, was also used as a numerical and categorized variable, as normal or impaired [45,46]. The GDS scores were also converted into binary outcomes to reflect the presence (i.e. scores of 6 or more) or absence (i.e. scores of 5 or less) of relevant symptoms for clinical depression [46]. The Athens Insomnia Scale (AIS) score was considered as a numerical and categorized variable: normal or impaired [47].

The Barthel Index assesses the ability to perform activities of daily living by measuring the level of independence with 10 items, with a score range of 0–100 [49].

### 2.4 Statistical analysis

Descriptive statistical analyses were performed. Kolmogorov-Smirnov tests were performed to determine which variables fit the normal distribution; most of the statistical analyses performed were nonparametric. Both parametric (Pearson) and nonparametric (Spearman) bivariate correlations were calculated. For descriptive statistics, we calculated the frequency distribution for the descriptive study of qualitative variables. For quantitative (discrete) variables, measures of central tendency (arithmetic mean), dispersion (standard deviation (SD) and the range of values were obtained. Mann-Whitney test was used to compare differences between two independent groups when the dependent variable is either ordinal or continuous (for example to compared data between women who received previous chemotherapy versus those did not). The Friedman Test was used to determine if data recorded at baseline (before starting AROi treatment), 6 months under AROi and 12 moths under AROi from the same group are different from each other on a skewed variable of interest. Friedman U-tests were performed. The statistical relationship between two categorical variables was studied using Pearson's Chi-square test. For the analysis of two-way randomized block designs where the response variable can have only two possible outcomes (coded as 0 and 1), Cochran's Q test was used to determine if there are differences on a dichotomous dependent variable (presence or not of insomnia or depression based on cut-off score) between three measurements (baseline, under 6 and 12 months of treatment with AROi). Multivariate statistical analysis including logistic regression analyses was used to predict the probabilities of the different possible outcomes of a categorically distributed dependent variable, given a set of independent variables e.g. the associations between clinical endpoints relevant to worsening depression and insomnia such as patients' age, previous chemotherapy administration, cancer stage, BMI, Charlson comorbidity index and polypharmacy. Odds ratios (ORs), 95% confidence intervals (95% CIs) and statistical significance (p) were estimated for each predictor variable. All statistical tests were considered statistically significant at the p < 0.05 level. The analyses were carried out with the IBM Statistical Package for Social Sciences SPSS software package (version 26.0; SPSS, Inc., Chicago, IL, USA).

## 3 Results

### 3.1 Clinical and socio-demographic characteristics of the study sample

Fifty-six breast cancer patients were included at baseline and were followed up for 12 months under AROi treatment. All the patients had tumors with estrogen and progesterone positive staining in the histological tissue analysis. The patients presented TNM disease stages, corresponding to local stage I (30 (53.6%)), or early regional stage IIA (16(28.6%)), IIB (7(12.5%)), or late regional stage IIIA (3(5.4%)). None of the patients presented distant metastases. All patients had received surgical treatment, with either conservative surgery 49 (87.9%) or mastectomy 7 (12.5%). Some of them received adjuvant chemotherapy 17 (30.4%) and radiotherapy [48] (85.7%) as per the protocol of their adjuvant treatment. One month after the last radiotherapy session, all the women started adjuvant hormonal treatment with aromatase inhibitors (anastrozole or letrozole).

The mean age of the participants was 66.43 ± 1.1 years (SEM) (range 51–84), and all of them were medically diagnosed as being postmenopausal before AROi administration. The participants were mostly married 26 (46.4%) or divorced 14 (25.0%), widows 12 (21.4%), and 4 (7.1%) were single. As for the type of histology, most patients had ductal carcinoma 55 (98.1%) while one (1.8%) had papillary carcinoma.

Mean estrogen receptor staining was 92.5 ± 1.4 (percentage ± SEM) (range 40–100) versus 61.2 ± 4.7 (percentage ± SEM) (range 0–100) for progestogens. The mean value for the proliferation marker ki67 was 15.5 ± 1.9 (percentage ±SEM) (range 1–60). The mean number of daily drugs was 3.1 ± 0.29 (range 0–11). The Charlson Index yielded a mean score of 2.4 ± 0.09 (SEM) (range 2–5). The mean body mass index (BMI) of the participants was 28.9 ± 0.75 kg/m2 (SEM) (range 18.6–45.1).

### 3.2 Analysis of cognitive functions, depressive symptoms, sleep quality and basic activities of daily living

Table 1 presents the mean score of each scale at baseline and after 6 and 12 months under AROi treatment. There were no significant differences for cognitive functions during treatment in the MMSE score or in any cognitive subdomain (time orientation, fixing-recall, attention-calculation, delayed recall and language). The spatial orientation subdimension improved under AROi treatment (p = 0.007, Friedman test).

**Table 1:** Means core and comparisons of psychological alterations in breast cancer patients under adjuvant AROi treatment.

Cognitive functions	Basaline Mean score ± SEM	6 months under AROi treatment Mean score ± SEM	12 months under AROi treatment Mean score ± SEM	p values
Time orientation	4.85 ± 0.048	4.66 ± 0.105	4.77 ± 0.107	0.138
Spatial orientation	4.89 ± 0.050	4.98 ± 0.20	4.98 ± 0.023	0.007
Fixing-recall	3.00 ± 0.00	3.00 ± 0.00	3.00 ± 0.00	1.000
Attention – calculation	4.42 ± 0.155	4.46 ± 0.149	4.48 ± 0.155	0.428
Delayed recall	2.64 ± 0.095	2.50 ± 0.115	2.43 ± 0.119	0.804
Language	8.63 ± 0.116	8.70 ± 0.087	8.64 ± 0.092	0.516
Total cogntive function score	28.46 ± 0.269	28.30 ± 0.324	28.36 ± 0.291	0.777
Depressive symptoms	2.1 ± 0.3	2.8 ± 0.4	2.7 ± 0.5	0.851
Sleep quality	2.9 ± 0.4	2.8 ± 0.4	3.2 ± 0.5	0.198
Barthel index	98.0 ± 3.1	98.4 ± 3.0	95.6 ± 8.2	0.049

**Fig. 1:**
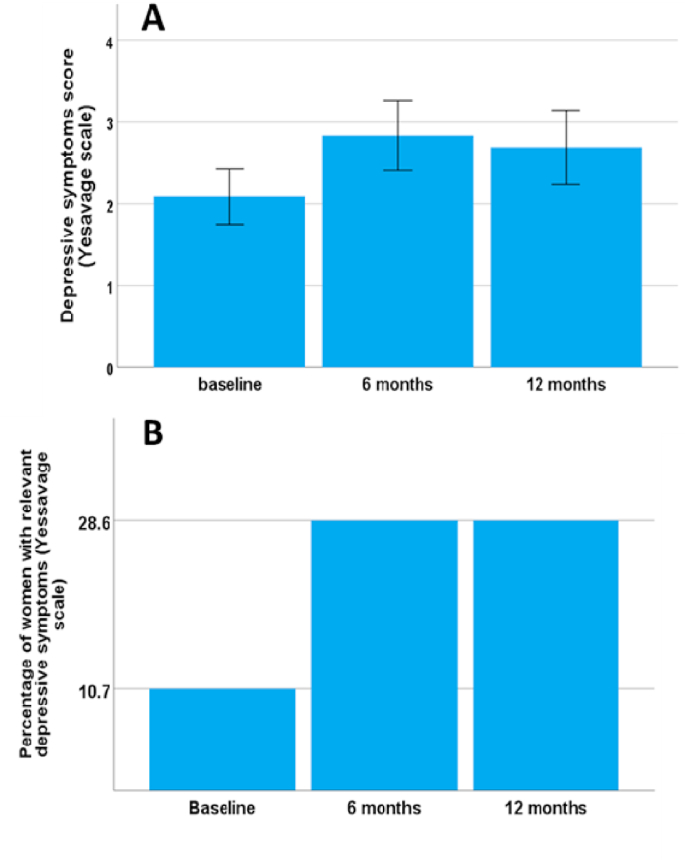
(A) Mean score on the GDS scale and (B) percentage of patients with clinically relevant depression (cut-off ≥ 6) at baseline, and after 6 and 12 months under AROi treatment.

The MMSE scores obtained indicated a greater mild cognitive impairment before starting adjuvant hormone treatment (16.1%), while there was an improvement (10.7%) after one year of treatment, although this was not statistically significant. The scores on the GDS scale for depression are shown in Fig. 1. Although in overall terms there was no significant change in the mean scores of depressive symptoms over time (baseline 2.1 ± 0.3; after 6 months under AROi treatment 2.8 ± 0.4; after 12 months under AROi treatment 2.7 ± 0.5; p = 0.29; Fig. 1A) when dichotomizing values with the cut-off ≥ 6 we observed a statistical increase (p = 0.02) in the percentage of patients with clinical relevant symptoms of depression (Fig. 1B) (baseline 10.7% of the study sample; after both 6 and 12 months under AROi treatment 28.6% of the study sample).

**Fig. 2:**
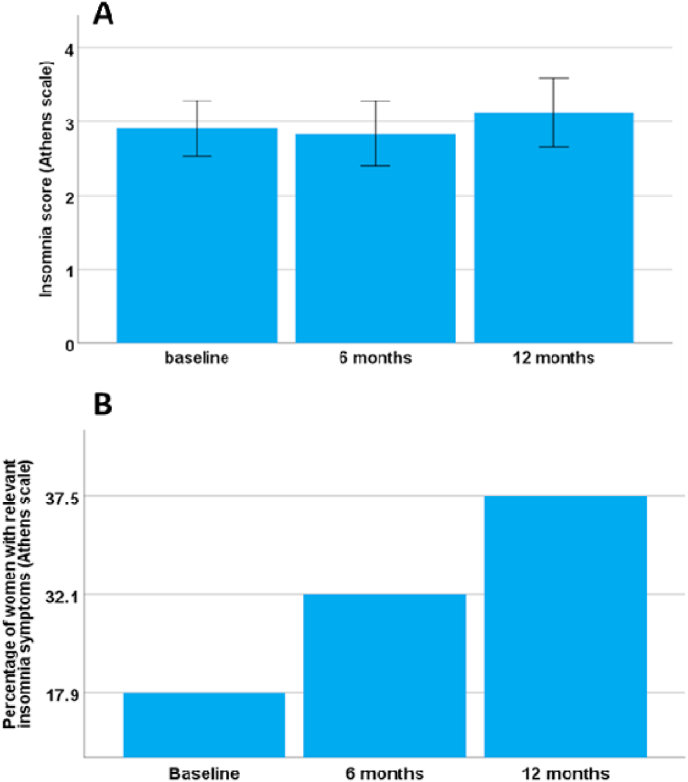
(A) Mean Athens scale score and (B) percentage of patients with clinically relevant depression (cut-off ≥ 6) at baseline, and after 6 and 12 months under AROi treatment.

The scores on the Athens scale for insomnia are shown in Fig. 2. Although in overall terms there was no significant change in the mean scores of insomnia symptoms over time (baseline 2.9 ± 0.4; after 6 months under AROi treatment 2.8 ± 0.4; after 12 months under AROi treatment 3.1 ± 0.5; p = 0.72; Fig. 2A) when dichotomizing values with the cut-off ≥ 6 we observed a statistical (p = 0.001) increase in the percentage of patients with clinically relevant symptoms of depression (Fig. 2B) (baseline 17.9% of the study sample; after 6 months 32.1% and 12 months under AROi treatment 37.5% of the study sample).

### 3.3 Relationship between the variables measured in the psychogeriatric evaluation

Correlations were calculated to determine the interaction between the quantitative variables. No significant associations were found between cognitive function (MMSE score) and depressive symptoms (Yesavage scale) at any time point (p = 0.070 at baseline, p = 0.405 after 6 months under AROi treatment and p = 0.381 after 12 months under AROi treatment). No significant associations were found between cognitive function (MMSE score) and insomnia symptoms (Athens scale) at any time point (p = 0.274 at baseline, p = 0.932 after 6 months under AROi treatment and p = 0.933 after 12 months under AROi treatment). In contrast, cognitive functions were consistently associated with the ability to perform the basic activities of daily life at each time point (Barthel index) (p = 0.001 at baseline, p = 0.017 after 6 months under AROi treatment and p = 0.045 after 12 months under AROi treatment). Statistically significant associations were found between the Athens and Yesavage scales at baseline (p = 0.011, Spearman correlation) and after 6 months under AROi treatment (p = 0.001, Spearman correlation) but not a 12 months under AROi treatment ((p = 0.389, Spearman correlation).

### 3.4 Associations between depressive and sleep quality with clinical variables related with other clinical variables

Next we analyzed if age, the presence of previously administered chemotherapy and body mass index, or the comorbidities index has a significant role in the significant increase in the percentage of patients with relevant depressive symptoms. No significant effect of age, previously chemotherapy administration or comorbidity index or IMC was observed for depressive symptoms (p > 0.05 for each comparison e.g. baseline, 6 months and 12 months under AROi treatment). We also analyzed any association with histological parameters of breast tumors such as estrogen and progestogen-positive staining, HER-2 and KI67 staining. For dichotomic depressive symptoms, the only significant results were found at baseline with ki67 values (women with breast tumors with higher ki67 values had a significant probability of having relevant clinical symptoms of depression, p = 0.028). The association with ki67 was not significant after 6 and 12 months under AROi treatment (p > 0.05 in both cases). For depressive symptoms no significant effect of age, previous chemotherapy administration or comorbidity index or IMC was observed (p > 0.05 for each comparison e.g. baseline, 6 months and 12 months under AROi treatment). The associations between dichotomized relevant insomnia symptoms and histological tumor markers revealed that at baseline, women with relevant insomnia symptoms have significant higher ki67 values compared to women with fewer or no insomnia symptoms (p = 0.007). This effect remains significant after 6 months under AROi treatment (p = 0.035).

Logistic regression was performed to determine if there was a significant relationship with the clinical variables also in multivariate analyses. The variables analyzed were age, number of daily drugs, Charlson comorbidity index, polypharmacy, previous chemotherapy administration, cancer stage, percentage of staining of progesterone and estrogen receptors, Ki67 in breast tissue, presence or not of depression and insomnia at baseline (before receiving AROi treatment). A significant association was found between the dichotomous dependent variable Yasavage using the cut-off score for the presence of depression (dichotomized as improved or stable = 0; worsened = 1 at follow-up) and the explanatory variable previous chemotherapy administration (p = 0.047), OR = 3.66 (95%CI 1.01–13.2), being a significant risk factor. Likewise, there was a significant association with age (p = 0.038), OR = 0.85 (95% CI 0.73–0.99) (younger women had higher risk to worsen depressive symptoms) and a tendency for cancer stage (p = 0.053), OR = 2.00 (95% CI 0.66–6.06) (patients with higher stage of breast cancer had higher risk to worsen depressive symptoms). In contrast the other variables there were no significantly associated with the risk of depression at follow-up (data not shown). In contrast, a significant association was found between the dichotomous dependent variable Athens scale using the cut-off score for the presence of insomnia (dichotomized as improved or stable = 0; worsened = 1 at follow-up) and the explanatory variable previous chemotherapy administration (p = 0.03), OR = 9.85 (95%CI 1.13–85.8), being a significant risk factor for insomnia. In contrast the other variables there were no significantly associated with the risk of insomnia at follow-up (data not shown).

## 4 Discussion

The effects of AROi treatment on psychological alterations have been analyzed with long follow-up periods (12 months) as in the present study in very few cases in the literature [[50], [51], [52], [53]] and focused on pain and quality of life [50] or treatment-emergent symptoms detected by worsening patient-reported outcome scores [52]. Depressive and cognitive functions were prospectively analyzed in one large study but they were secondary outcomes to make comparisons with tamoxifene treatment [51]. For cognitive functions, the consequences of treatment with AROi have been evaluated in multiple studies with discordant results. In our study, no significant effect on cognitive function over time were detected in patients with localized breast cancer treated with aromatase inhibitors. Several studies that collected data on the evolution of cognitive function in chemotherapy-naïve breast cancer patients treated with aromatase inhibitor and a control arm show a disparity of results in terms of the impact on cognitive function [[52], [53], [54], [55], [56], [57]]. These conflicting results may be due to different study participant selection methods with both premenopausal and postmenopausal populations, assessment of the effects of aromatase inhibitors compared to tamoxifen with no comparative control group, differences in cognitive reassessment times, and the use of different cognitive function measurement tools. In a study published by Bender et al. [55], the administration of the AROi anastrozole was associated with a worsening of significant executive function, and with a worsening of visual working memory, as well as of concentration compared to the control group. The instrument used in our study, the MMSE used for the screening of cognitive impairment in primary care, did not measure working memory or executive function, and as such we cannot rule out the possibility those cognitive subdomains could have been impaired by AROi and further studies are warranted. However, other studies showed that there was no alteration in the results of neuropsychological tests over time after the start of adjuvant hormonal treatment with aromatase inhibitors [56,57]. We cannot rule out that longer follow-up studies (>1 year) under AROi treatment could detected cognitive impairment [54,55,58]. Regarding depressive symptoms, it has been reported that up to 25%–30% of women with breast cancer report these symptoms at some point since diagnosis [59,60], and identifying a set of factors that are linked to depression in patients with early-stage breast cancer on adjuvant treatment is challenging. The risk of depression may be associated with the cancer, also with the severity of the disease, with the treatment itself (due to its adverse effects, as is the case of depression associated with peripheral neuropathy induced by adjuvant chemotherapy [28,61,62]). We detected an increase in the percentage of patients with relevant burden of depressive symptoms increased under AROi treatment. Previous studies have postulated conflicting results regarding depressive disorders among breast cancer survivors who received adjuvant therapies including third-generation AROi [63,64]. In a population-based study with a defined breast cancer cohort investigating the risk of depressive disorders in breast cancer patients who received adjuvant therapies, of the total 36,586 patients, 1342 (3.7%) were diagnosed with depressive disorders and Cox multivariable proportional hazards analysis showed AROi treatment was an independent risk factor for developing depressive disorders [63]. However, there are also studies that show that patients treated with AROi present an improvement in symptoms of anxiety and depression, such as the study published by Martino et al. [65], who examined the presence of depression in breast cancer patients and demonstrated that after 6 months of AROi treatment, breast cancer survivors showed significantly reduced anxious and depressive symptoms and significantly higher perceived quality of life for physical and mental components compared with controls. In our study, patients were analyzed for the presence of depressive symptoms shortly after being treated surgically and with little time since diagnosis, which also favors the finding of depressive symptoms [66].

Sleep disturbances are also frequent in patients treated with AROi as adjuvant treatment for localized breast cancer. According to a study of 413 women with early-stage breast cancer receiving treatment with AROi (having been previously treated with surgery and radiotherapy) [42], complaints or symptoms of insomnia exceeded 50%, with 19% of women exceeding the validated threshold for clinically significant insomnia. Our study showed that the percentage of patients with a relevant burden of insomnia symptoms increased under AROi treatment. Only one study has been performed on this subject, and our study therefore supports the influence of AROi treatment in patients with high symptoms of insomnia at baseline. There are several prospective studies in the literature reporting a high incidence of insomnia, but these studies do not use a validated scale to analyze the diagnosis of clinically relevant insomnia at the beginning of taking the drug, nor do they evaluate its evolution over time [67,68]. There are few studies prospectively assessing the relationship between the initiation of adjuvant AROi therapy and the onset or worsening of symptoms related to insomnia [69]. In a prospective study with a relatively small sample size of 23 women with early-stage breast cancer who were candidates for initiating treatment with aromatase inhibitors [70], no association could be identified between discontinuation of AROi and changes in sleep quality assessment, in sleep disturbance measured by actigraphy, or in reported sleep symptoms (reported by patients according to Common Fund's Patient-Reported Outcomes Measurement Information System (PROMIS) questionnaires) presented after 3 months of AROi treatment. In another prospective study in women evaluated prior to the start of AROi for adjuvant treatment of breast cancer, after the first year of hormonal treatment, women with poor sleep quality prior to the start of AROi were more likely to discontinue due to treatment toxicity than women with good initial sleep quality [71]. In our study, women who already had insomnia as measured by the Athenas scale prior to the initiation of AI were also those who had insomnia after the initiation of hormone treatment and the incidence increased as we found that 17.9% of the patients presented relevant burden symptoms of insomnia at baseline, and this proportion represented 37.5% of the women treated after one year.

To date, the hypothesis that aromatase inhibitors might have an adverse impact on cognitive function had led to mixed results [72,73]. This study did not detect any significant relationship between cognitive impairment and depression, nor between cognitive impairment and insomnia were found, suggesting that these alterations when they take place do not share the same mechanisms under AROi. The interaction between depression and insomnia symptoms was only observed in the bivariate analyses suggesting a partial overlapping between these psychological alterations. In contrast, the multivariate analysis showed that some clinical factors are significantly associated with a higher probability of worsening of depressive symptoms and insomnia during AROi treatment e.g. previous chemotherapy administration, and younger age. These results have an important implication for clinical practice in a multidisciplinary team and suggest the subgroup of younger patients and/or those who received adjuvant chemotherapy could be more vulnerable to these alterations [74,75] and may require more social and psychological support [76]. The association between insomnia increase under AROi and previous treatment with chemotherapy has been reported in one study and there might be a role of somatic symptoms related to the adverse effects of cancer treatments [69] but clearly need to be explored in future studies.

### 4.1 Limitations

This study has several important limitations that should be considered when interpreting its findings. These include the study design, given that it is an observational uncontrolled study. It should be noted that the research was carried out in a single hospital center in Spain, with a small number of participants, and the findings may therefore not be generalizable. Studies with more participants are required to confirm our results. In addition, our study did not assess the participation of patients who had received psychotherapy or psychological counseling before or during AROi treatment. Finally, the short observation period of 12 months did not allow us to detect how the variables explored would change over time in the longer term, in relation to hormonal therapy prescribed for a long period of time. Larger samples analyzed in prospective studies are required to better understand and quantify the factors influencing the psychological alterations induced by AROi. The findings of this study suggest that carrying out a screening to identify the appearance of depressive symptoms and sleep problems in patients treated with AROi would be useful, since they seem to worsen after the initiation of AROi.

## Author contributions

Conceptualization J.G.-S., M.D.T. and O.C.; methodology J.G.-S., M.D.T. and O.C.; formal analysis J.G.-S. and M.A.M.-E.; investigation: J.G.-S., M.A.M.-E., O.C., M.D.T. and O.C.; writing—original draft preparation J.G.-S., M.A.M.-E., M.D.T. and O.C.; writing—review and editing J.G.-S., M.A.M.-E., M.D.T. and O.C. All authors have read and agreed to the published version of the manuscript.

## Funding

This research was funded by the 10.13039/501100003508University of Valencia, with grant number UV-19-INV_AE19.It is correct!

## Institutional review board statement

The present study was conducted in accordance with the Declaration of Helsinki, and was approved by the Ethics Committee of Dr. Peset University Hospital (Valencia, Spain) (protocol code 57/18, date of approval: July 25, 2018).

## Informed consent statement

Informed consent was obtained from all subjects involved in the study.

## Data availability statement

The data presented in this study are available on request from the corresponding author for scientific purposes.

## Declaration of competing interest

The authors declare that they have no known competing financial interests or personal relationships that could have appeared to influence the work reported in this paper.
